# A Case Report of Acute Compartment Syndrome

**DOI:** 10.21980/J87061

**Published:** 2024-04-30

**Authors:** Naomie Devico Marciano, Keneth Sarpong, Jonathan Smart

**Affiliations:** * University of California, Irvine, Department of Emergency Medicine, Orange, CA

## Abstract

Acute compartment syndrome (ACS) is a surgical emergency which requires prompt identification and intervention to prevent irreversible tissue damage. Here we present the case of a 64-year-old male with lower extremity tenderness following a crush injury. This patient presented to the emergency department (ED) more than 12 hours after the initial incident occurred and was found to have a firm right calf with decreased sensation and absent distal pulses on his right leg. The patient’s outer compartment pressure measured 32 mmHg. Because these findings were concerning for acute compartment syndrome, emergent fasciotomies of the four compartments of the lower right leg were performed with improvement in neuromuscular compromise. Early identification of the condition permitted a prompt recovery for the patient who was discharged home on day five. This case report reviews the clinical presentation and interventional modalities and aims to provide new images to help visualize a diagnosis of ACS.

**Topics:**

Acute compartment syndrome, fasciotomy, intramuscular pressure.

[Fig f1-jetem-9-2-v1] [Fig f2-jetem-9-2-v1] [Fig f3-jetem-9-2-v1]

**Figure f1-jetem-9-2-v1:**
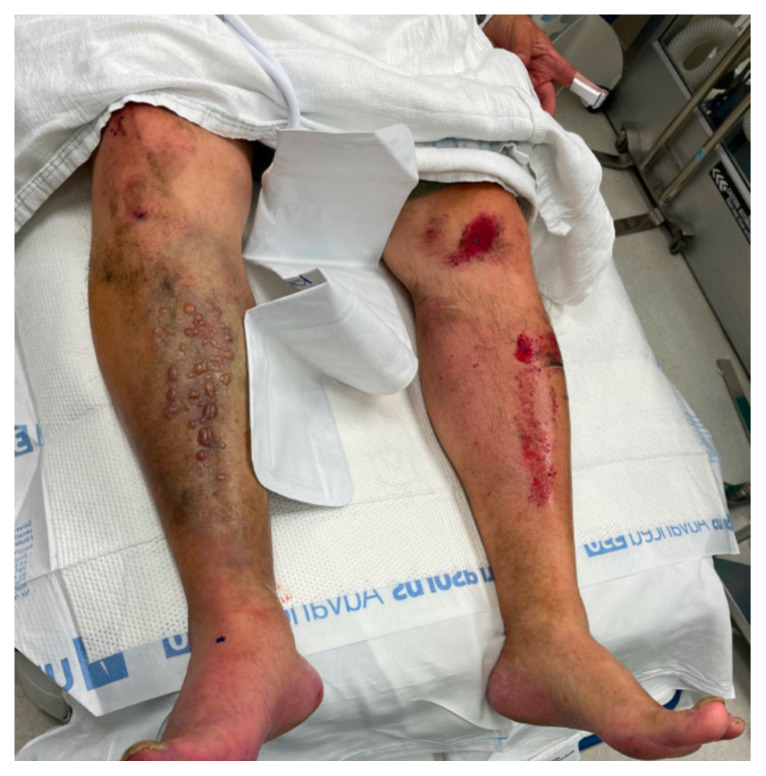


**Figure f2-jetem-9-2-v1:**
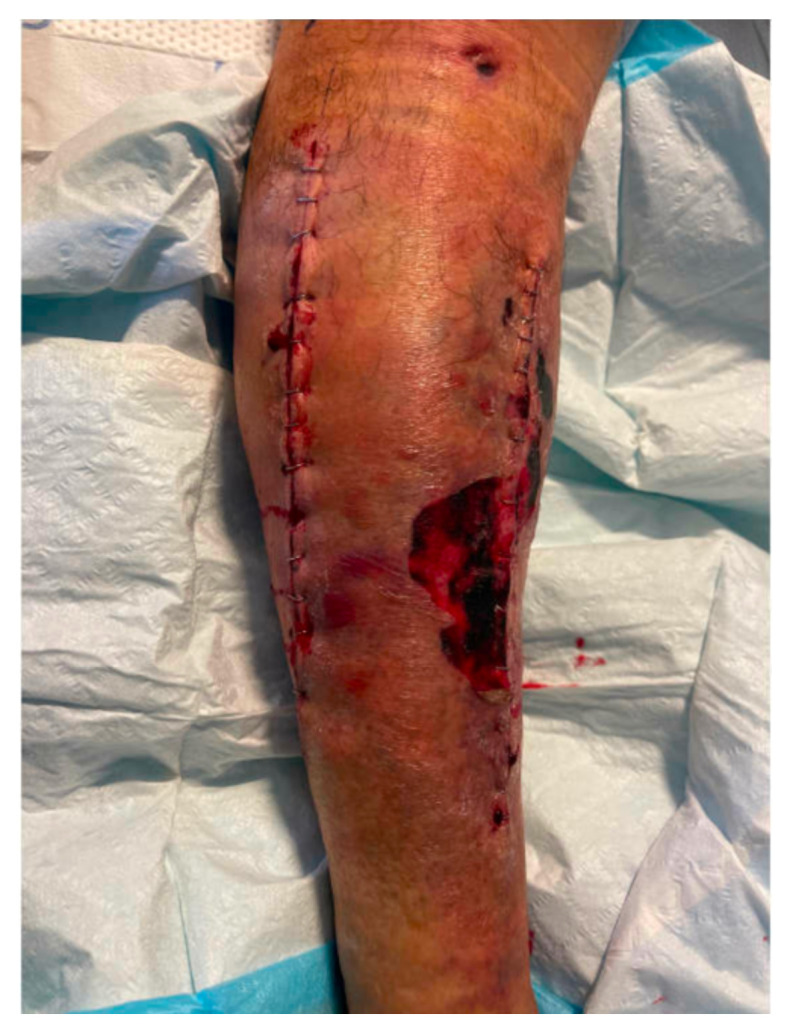


**Figure f3-jetem-9-2-v1:**
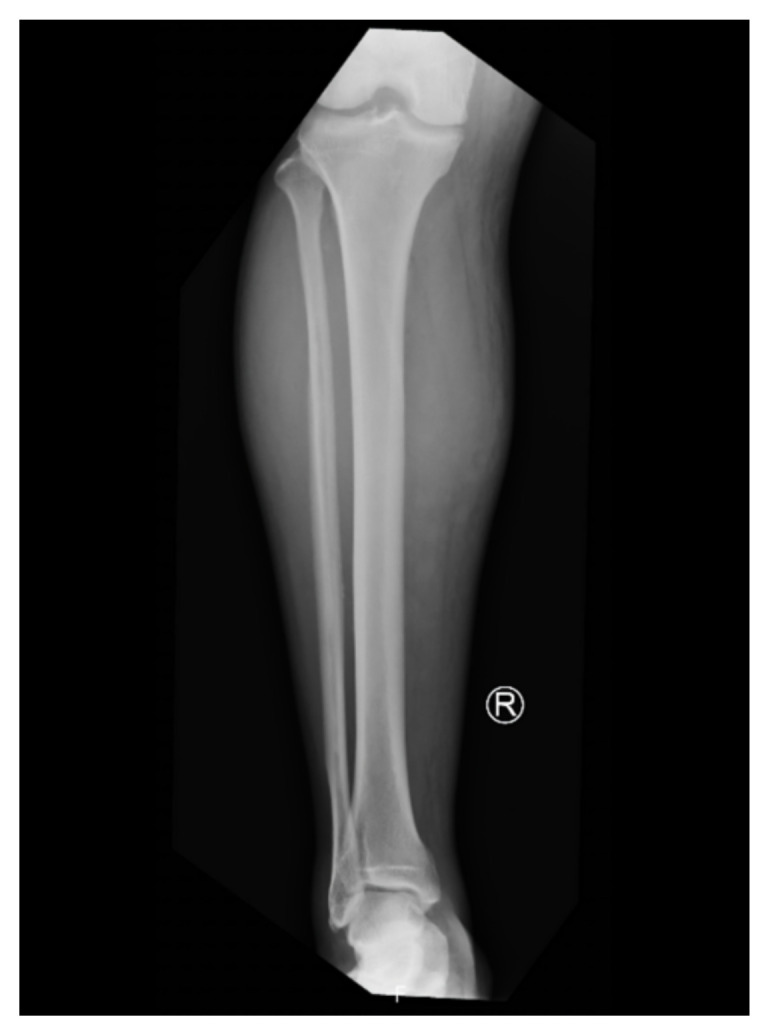


## Brief introduction

Acute compartment syndrome (ACS) arises from elevated pressure within a confined osteofascial compartment, leading to significant pain. This heightened pressure reduces muscle perfusion, causing subsequent decrease in oxygen delivery to affected tissues.^1^,^2^ ACS can progress rapidly, constituting a surgical emergency with considerable morbidity risk due to potential ischemia and subsequent necrosis if the neurovascular deficit is not treated immediately.^3^,^4^ Compartment syndrome most frequently occurs in the anterior compartment of the leg with tibial diaphyseal fracture causing an estimated 36% of ACS.^5^ However, ACS may develop in various anatomical compartments, such as the arm, forearm, hand, thigh, gluteus, lower leg, foot, or abdomen. 6–8

Most cases of ACS (75%) are linked to long bone fractures, particularly the tibia, where ACS occurs in 1–10% of cases.^1^ Treatment for both closed and open fractures heightens ACS risk, necessitating vigilant monitoring. Non-fracture causes include forceful trauma, severe burns, and penetrating injuries. Vascular injury, particularly, is significant in ACS because bleeding raises compartment pressure, leading to muscle ischemia and subsequent reperfusion injury.^9^

Less common nontraumatic causes of ACS include hematologic disorders, anticoagulant use, nephrotic syndrome (lowering serum osmolarity), prolonged limb compression, revascularization procedures, or Group A streptococcus muscle infection. 10

Incidence is ten times higher in males than in females.^2^ Commonly ACS develops in younger patients (under 35 years of age), with a higher incidence in males, likely due to larger inter-compartmental muscle mass as well as this population’s increased risk of involvement in high energy trauma. 1

Clinically, the symptoms of ACS and signs found on examination may progress rapidly. Diagnosis relies on measuring compartment pressure and monitoring for changes over time. A focused neuromuscular exam is crucial, and special attention should be given to the classic “6 Ps” which include pain, pallor, poikilothermia, pulselessness, paralysis and paresthesia. Notably, disproportionate pain is an early finding.^8^ Laboratory studies are not used for diagnosis; however, serum creatinine kinase (CK) could be tracked due to potential for rhabdomyolysis.

Here, we describe the case of a 64-year-old male diagnosed with acute compartment syndrome following a crush injury. We further discuss clinical findings, workup, current recommendations for treatment, and provide new images to help better visualize this diagnosis. Patient-written consent for medical photography was obtained during the patient’s admission.

## Presenting concerns and clinical findings

A 64-year-old male with a past medical history of hypertension and diabetes was transferred to our ED due to lack of palpable pulses on the right leg following a crush injury. The patient reported having pulled over to the side of the road the previous day. After having stepped out of the vehicle the car rolled over on his legs. His lower extremities were trapped beneath the car for an estimated 15 minutes. At the time of the first ED presentation at an outside hospital, the patient was reportedly intoxicated and left against medical advice. However, the patient experienced continually worsening pain at home, returned to the outside hospital ED, and was transferred to our ED as a trauma patient. Vital signs were within normal limits except a mildly elevated blood pressure at 146/82 mmHg. The patient described his pain as dull with diffuse radiation and noted that it was associated with diffuse numbness and tingling of the right lower extremity below the level of the knee.

## Significant findings

Inspection of the extremity revealed significant swelling with dark discoloration and multiple bullae (pre-operative photograph). Furthermore, notable swelling of the right foot was noted, which felt cold to palpation. The right calf was firm and tender to palpation, with decreased sensation. No motor deficits were noted. Posterior tibial (PT) and dorsalis pedis (DP) pulses were not palpable on the right but the DP doppler pulse was obtained. A compartment pressure of 32 mmHg was measured.

Radiographs of pelvis, bilateral knees, tibia, fibula, and feet demonstrated no fractures or dislocations. The bilateral tibia and fibula X-ray revealed soft tissue swelling in the proximal legs, particularly evident in the right leg’s AP view, which also showed numerous ovoid radiodensities in the anterior compartment, likely related to soft tissue injury. On admission, the patient’s serum lactic acid was elevated (3.1 mmol/L), and creatinine kinase was elevated as well (240 U/L). These laboratory abnormalities were likely due to muscle injuries but did not meet criteria for rhabdomyolysis.

Post operative images are also provided demonstrating the patients’ four compartment fasciotomies which were loosely closed using staples.

## Patient course

Given the concern for acute compartment syndrome, trauma surgery was consulted, and the patient was taken emergently to the operating room for four compartment fasciotomies of the lower right leg. During the first post-operative day, the patient complained of increasing pain in the left lower extremity; however, compartments in that extremity remained soft to palpation and without neurologic deficits. Pulses were also palpable. The incision on the right lower extremity continued to show bleeding despite pressure dressing having been placed. To control the bleeding, several staples were removed to expose the wound and bleeding muscle was visualized and cauterized.

By the third post-operative day, bleeding had stopped. During his hospital course, the patient did not develop neurological deficits over his lower extremity. The patient was discharged home on hospital day five after physical therapy and wound care teaching. At last contact, the patient was unable to follow up appointments at our institution since he was being treated elsewhere for wound debridement and poor progression of his right lower extremity wound. Over the next 6 months, the patient developed complex regional pain syndrome, initially treated with physical therapy and gabapentin, then switched to pregabalin due to tolerance. However, conservative measures failed, prompting trigger point injections in the gastrocnemius and soleus muscle groups.

## Discussion

The diagnosis of ACS relies heavily on clinical evaluation. Key indicators include rapidly worsening symptoms, such as “pain out of proportion to the injury,” along with a tense compartment exhibiting a firm, “wood-like” texture.^1^ Additionally, classical signs such as pulselessness, paresthesia, paralysis, and pallor are observed.^1^ If compartment syndrome is suspected, immediate surgical consultation is required. However, in unconscious patients or those with a history of sensorineural loss, history and physical examination alone may not suffice.^11^

Measuring compartment pressure serves as a valuable supplementary test: a critical delta pressure of 30 mmHg or less, indicating the difference between diastolic pressure and compartment pressure, is often employed as the diagnostic threshold for ACS.^4^,^12^ However, debates in the literature exist regarding the absolute threshold of 30mmHg for the delta pressure, with advocates for higher and lower cutoffs.^11^ Additionally, tissue and individual responses to elevated compartment pressure may vary.^11^,^13^ Importantly, a single pressure measurement does not estimate ischemia degree accurately. Technical errors and pressure variations based on measurement location further diminish the specificity of delta pressure testing.^3^,^11^,^14^

The pathophysiology of ACS can be attributed to cellular anoxia, muscle ischemia and subsequent tissue death.^15^ As pressure increases in the affected compartment, local tissue perfusion is restricted by a reduced arteriovenous pressure gradient which when prolonged leads to cellular anoxia. The lack of oxygen to tissue can lead to damage of both muscular and nervous tissue. Further propagation of the damage occurs as capillary blood flow decreases, enhancing blood vessel permeability and further increasing internal pressure.

Perfusion is typically compromised when pressure approaches 10–30 mmHg of diastolic pressure while nerve conduction is compromised when the compartment pressure becomes greater than 30 mmHg.^16^ However, this test provides low specificity, and determining an exact value for this specificity from the literature proves elusive.^9^,^14^ Nonetheless, management is often guided by a patient’s overall clinical presentation.^11^

Imaging studies typically do not contribute to diagnosing compartment syndrome but may aid in ruling out alternative etiologies; X-rays are the preferred initial imaging for evaluating anterior leg pain.^17^

Prompt recognition and treatment of ACS is critical because the damage often becomes irreversible within six hours and may result in long term morbidity because prolonged ischemia may cause liquefactive necrosis of the muscles or even mortality.^8^

Fasciotomy to decompress all involved compartments is the treatment of choice for ACS; however, hyperbaric oxygen therapy has also been described as an adjunct treatment.^18^,^19^ Other treatments include the prevention of hypotension and keeping the extremity at the level of the heart to prevent hypoperfusion. Prognosis is highly variable and relies on how quickly fasciotomy is performed to restore perfusion to affected tissue.^9^

The fasciotomy procedure entails making a large incision to decompress all compartments of the affected extremity.^20^,^21^ In the case of our patient’s lower leg, this involved decompression of the anterior, lateral and posterior superficial and deep compartments. Typically performed in the operating room under general or regional anesthesia, bedside fasciotomy under conscious sedation and local anesthesia should be considered in emergent.^22^ Studies suggest that bedside fasciotomy is a safe and effective option for patients with delayed presentations or in scenarios where significant delays in performing the procedure are anticipated.^21^ Of note, in the case of delayed presentation, the risk of reperfusion injury should also be considered.

Data has shown that when surgical procedure is done within the first six hours of onset, limbs can recover 100% of their function. However, this decreases to 2/3 of patients having normal limb function if it is done within 12 hours. When care is further delayed, amputation may be required.^20^ However, other studies reveal no significant difference in limb salvage rate when comparing early (<12h) to late (>12h) fasciotomy. ^23^ Post operative care includes physical therapy, wound care, pain management, and antibiotics if development of subsequent infection is suspected.^24^

In this case we present a 64-year-old man with ACS. Although rarer in older individuals, the crush injury placed him at higher risk. Prompt identification of the signs of ACS and rapid surgical intervention resulted in resolution of sensory deficits followed by a relatively short and uncomplicated hospital stay. Overall, this case highlights the importance of early identification and treatment of ACS because it is key in restoring function to the affected muscle.

## Supplementary Information






