# Supplementary figures and images for: A Case Report of Acute Compartment Syndrome
Source: J Educ Teach Emerg Med. 2024 Apr 30;9(2):V1–5. doi: 10.21980/J87061 (PMC11068313; doi:10.21980/J87061)

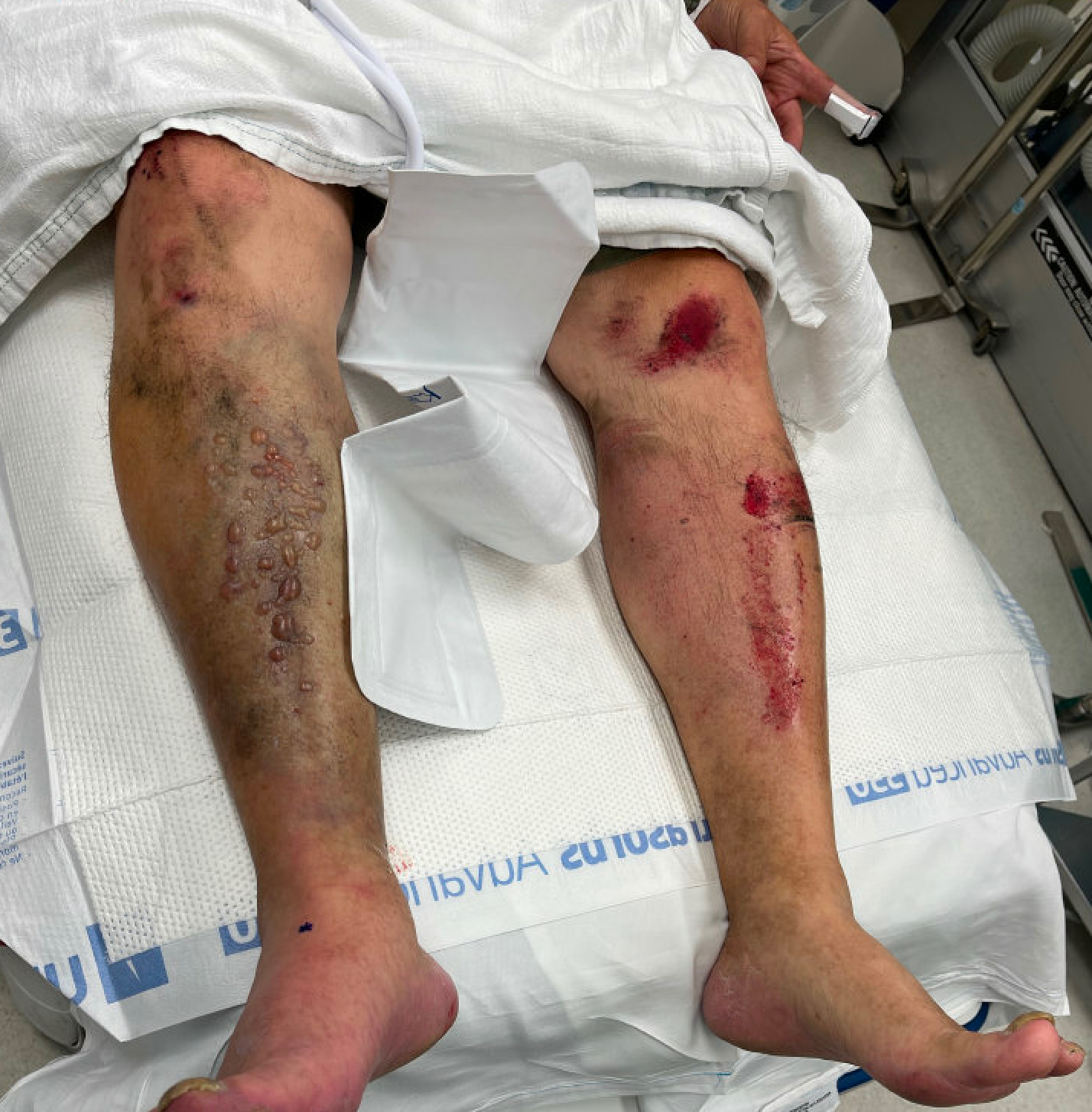

Supplement: Supplementary file 1 [file jetem-9-2-V1-supp1.jpeg]

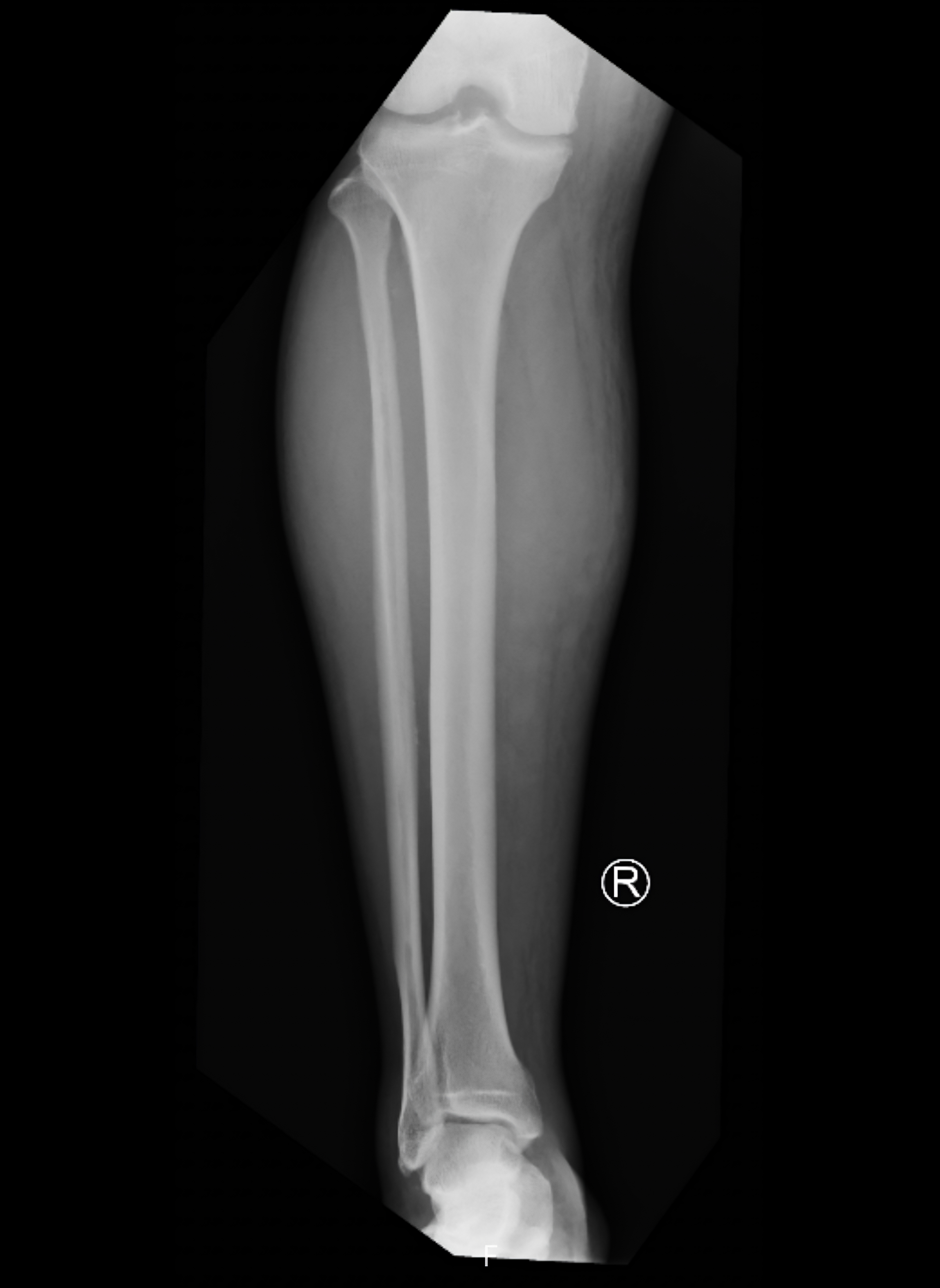

Supplement: Supplementary file 2 [file jetem-9-2-V1-supp2.jpeg]

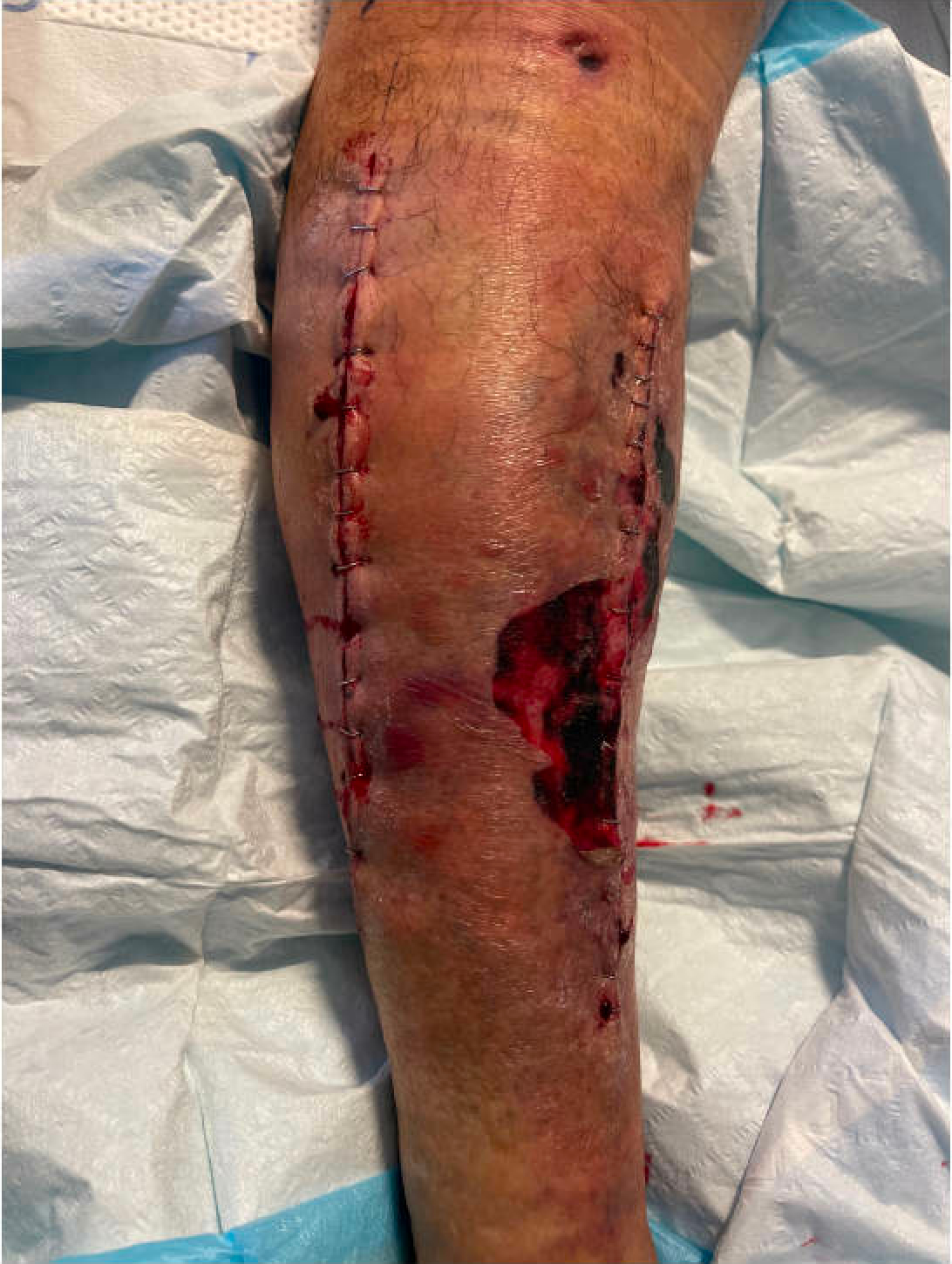

Supplement: Supplementary file 3 [file jetem-9-2-V1-supp3.jpeg]
